# Deep learning-based classification of adequate sonographic images for self-diagnosing deep vein thrombosis

**DOI:** 10.1371/journal.pone.0282747

**Published:** 2023-03-06

**Authors:** Yusuke Nakayama, Mitsuru Sato, Masashi Okamoto, Yohan Kondo, Manami Tamura, Yasuko Minagawa, Mieko Uchiyama, Yosuke Horii

**Affiliations:** 1 Department of Radiological Technology, Graduate School of Health Sciences, Niigata University, Niigata, Japan; 2 Department of Central Radiology, Niigata Cancer Center Hospital, Niigata, Japan; 3 Department of Radiology, Morita Comprehensive Diagnostic Imaging, Osaka, Japan; 4 Department of Nursing, Graduate School of Health Sciences, Niigata University, Niigata, Japan; 5 Department of Radiology, University Medical and Dental Hospital, Niigata University, Niigata, Japan; University of Montreal, CANADA

## Abstract

**Background:**

Pulmonary thromboembolism is a serious disease that often occurs in disaster victims evacuated to shelters. Deep vein thrombosis is the most common reason for pulmonary thromboembolism, and early prevention is important. Medical technicians often perform ultrasonography as part of mobile medical screenings of disaster victims but reaching all isolated and scattered shelters is difficult. Therefore, deep vein thrombosis medical screening methods that can be easily performed by anyone are needed. The purpose of this study was to develop a method to automatically identify cross-sectional images suitable for deep vein thrombosis diagnosis so disaster victims can self-assess their risk of deep vein thrombosis.

**Methods:**

Ultrasonographic images of the popliteal vein were acquired in 20 subjects using stationary and portable ultrasound diagnostic equipment. Images were obtained by frame split from video. Images were classified as “Satisfactory,” “Moderately satisfactory,” and “Unsatisfactory” according to the level of popliteal vein visualization. Fine-tuning and classification were performed using ResNet101, a deep learning model.

**Results:**

Acquiring images with portable ultrasound diagnostic equipment resulted in a classification accuracy of 0.76 and an area under the receiver operating characteristic curve of 0.89. Acquiring images with stationary ultrasound diagnostic equipment resulted in a classification accuracy of 0.73 and an area under the receiver operating characteristic curve of 0.88.

**Conclusion:**

A method for automatically identifying appropriate diagnostic cross-sectional ultrasonographic images of the popliteal vein was developed. This elemental technology is sufficiently accurate to automatically self-assess the risk of deep vein thrombosis by disaster victims.

## Introduction

Deep venous thrombosis (DVT) is characterized by the development of a thrombus in a deep vein that interrupts venous flow. Generally, the popliteal vein is a common site for DVT [1]. Typical symptoms include pain, swelling, redness, and edema [2]. Particularly, ultrasonography is recommended within 24 hours of suspected DVT [3]. Untreated DVT may induce pulmonary embolism (PE) [4]. The combination of DVT and PE is called venous thromboembolism (VTE). The estimated annual incidence of VTE in the United States is 1–2 per 1000 persons [5]. According to a systematic review of Sweden or the United States, 5.04 per 10,000 people develop DVT each year [6]. Therefore, VTE is a serious public health issue, and the early detection and treatment of VTE are important.

In recent years, VTEs have been reported in many post-earthquake victims. The incidence of DVT after the Great East Japan Earthquake in Ishinomaki in 2011 was 30.4% [7]. The incidence of DVT after the Kumamoto earthquake in 2016 was 10.6% [8]. Outside Japan, Tauqir et al. reported 3 cases of DVT (1 died of PE) in the Pakistan earthquake [9], and Groves et al reported 7 cases of DVT in the Nepal earthquake [10]. Thus, the incidence of DVT and PE after earthquakes is a worldwide concern. Therefore, mobile medical examinations in earthquake-affected areas of Japan include venous ultrasonography of the lower extremities [11]. However, quickly reaching all of the isolated and scattered shelters is difficult, although early detection is important. We hypothesized that a tool that allows non-medical victims to perform venous ultrasonography of the lower extremity and automatically measure their risk of developing DVT would be useful for early detection.

To enable victims to perform venous ultrasound examinations themselves, the following four elemental technologies are required: (1) assisted probe guidance, (2) automatic acquisition of cross-sectional images of veins, (3) automated evaluation of extracted vein regions, and (4) automated prediction of DVT risk based on the evaluation. First, the victim must operate the probe to properly visualize the popliteal vein. Therefore, a guiding function must be developed to inform the victim when the popliteal vein is clearly visualized in the image. The probe must be appropriately placed on the lower extremity to clearly display the popliteal vein. Subsequently, the popliteal vein image must be saved. A cross-sectional image of the vein must be acquired. Images are automatically updated in real-time during ultrasonography. Thus, once the popliteal vein is clearly visualized, the probe must not be moved before the image is saved. Therefore, elemental technique (2) is necessary. In addition, venous regions must be extracted from the obtained images and automatically measured (3). The results of this measurement can be used to automatically predict the risk of developing DVT (4).

In this study, deep learning was used to develop these elemental technologies. Recently, deep learning has been effectively applied to the analysis of ultrasonographic images [12, 13]. Furthermore, assessment of the risk of incident DVT by automated ultrasonography based on vein compressibility has been investigated [14]. Combined with these technologies, the system designed in this study to enable victims to assess their own risk of incident DVT is feasible. The purpose of this study is to develop and evaluate the elemental technologies necessary for the self-assessment of DVT risk, including (1) assisted guidance for the probe and (2) automatic acquisition of cross-sectional images of veins. The assisted guidance allows disaster victims to visually understand whether the probe is correctly put on the lower extremity, even if the victim has no experience in performing ultrasonography.

## Material and methods

### Overview of this study

**Fig 1:**
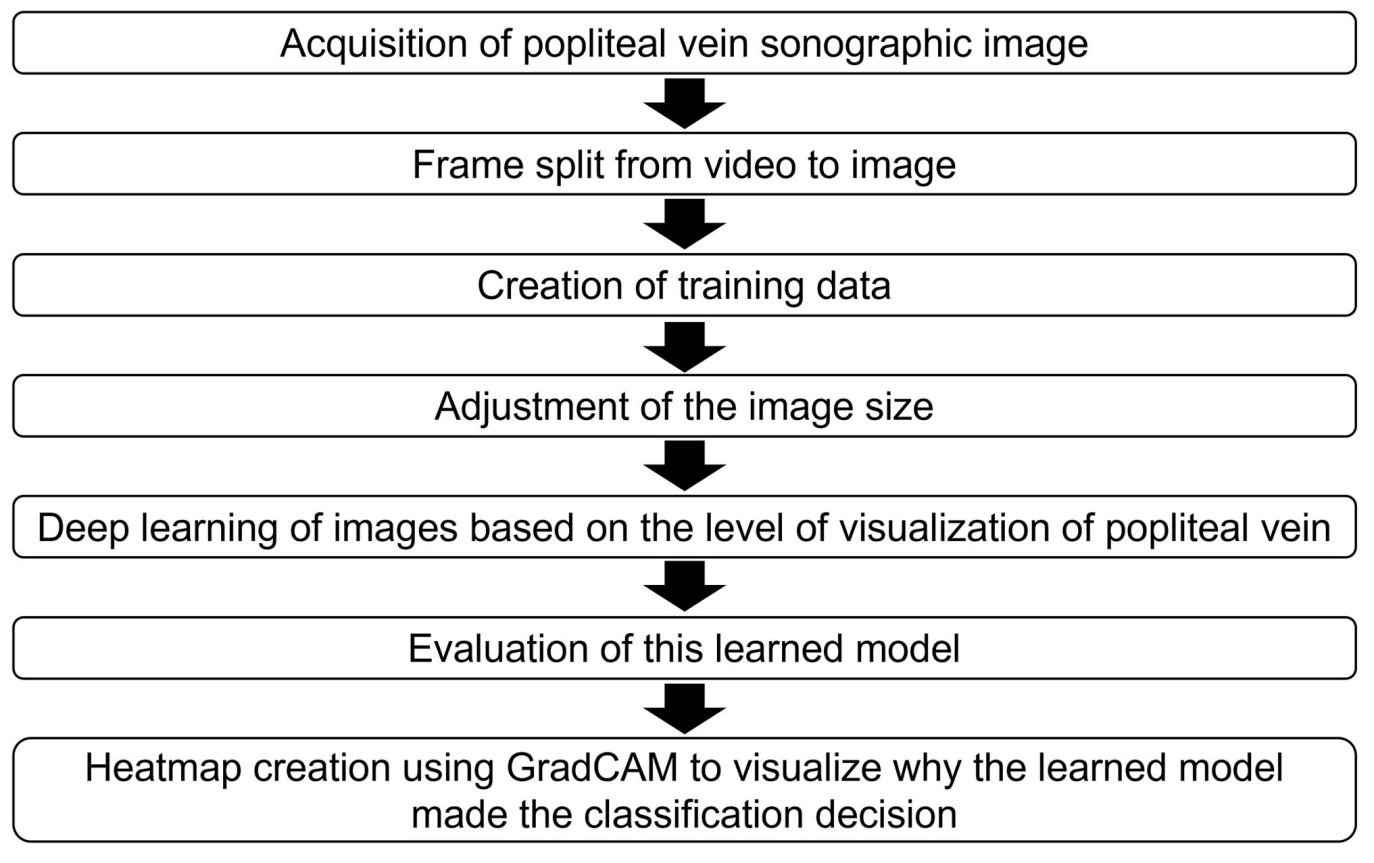
Overall scheme of the pre-processing and deep learning and its evaluation procedure.

The overall scheme of this study is shown in Fig 1. Venous ultrasonography of the lower extremities was performed. Subsequently, the acquired video was divided into frames, and the obtained images were used to generate the training data. The image size was adjusted to match the input size of the deep learning training data, and the Deep Convolution Neural Network (DCNN) was used for training and classification. The accuracy of the classification model was evaluated. Finally, a heatmap was created to confirm the focus of the DCNN as a basis for image classification. The details of each step are described below.

### Acquisition of the popliteal vein sonographic images

SNIMAGE HS1 (Konica Minolta Corp., Osaka, Japan) was employed as the stationary ultrasound diagnostic equipment, and Miruco (Nippon Sigmax Co., Ltd., Tokyo, Japan) was employed as the portable ultrasound diagnostic equipment. Short- and long-axis images were acquired using a linear probe in 20 subjects (11 males and 9 females) with healthy popliteal veins on both sides. The short-axis images were scanned vertically, shifted horizontally, and scanned in three lines. The long-axis images were scanned horizontally, shifted vertically, and scanned in two lines. Videos of venous compression were also acquired for both axes. Subjects were in the sitting position during imaging. Acquisition time was 3 to 4 minutes per person. All data were acquired by an experimental contributor who had no experience with ultrasound diagnostic equipment. The parameter settings for each device are shown in Table 1. The following parameters were used for the stationary ultrasonography equipment: Mode: venous; Frequency: 9 MHz; Gain: BG28; Depth of Field: 4 cm. For the portable ultrasonography, the following parameters were used: Mode: peripheral vascular; Frequency: 10 MHz; Gain: G48; Depth of Field: 4 cm. All procedures involving human participants were conducted in accordance with the ethical standards of the institutional and/or national research committee and the 1964 Declaration of Helsinki and its later amendments or comparable ethical standards. This study was accepted by research ethics committee in Niigata University (Acceptance number: 2020–0472). Informed consent was obtained from all subjects for the acquisition of lower extremity images using the ultrasound diagnostic equipment by written and verbal. However, the acquired images themselves were not authorized for release on the Internet.

**Table 1:** Description of ultrasound diagnosis equipment used in this study.

Setting	Stationary	Portable
Mode	Venous	Peripheral vascular
Frequency	9 MHz	10 MHz
Gain	BG28	G48
Depth of Field	4	4

### Images used for deep learning and classification

The ultrasonographic videos were divided into frames. The frame rate for the stationary ultrasound diagnostic equipment was 30 and 128,494 images were obtained from the ultrasonographic video. The frame rate of the portable ultrasound diagnostic equipment was 11 and 46,338 images were obtained from the ultrasonographic video.

**Fig 2:**
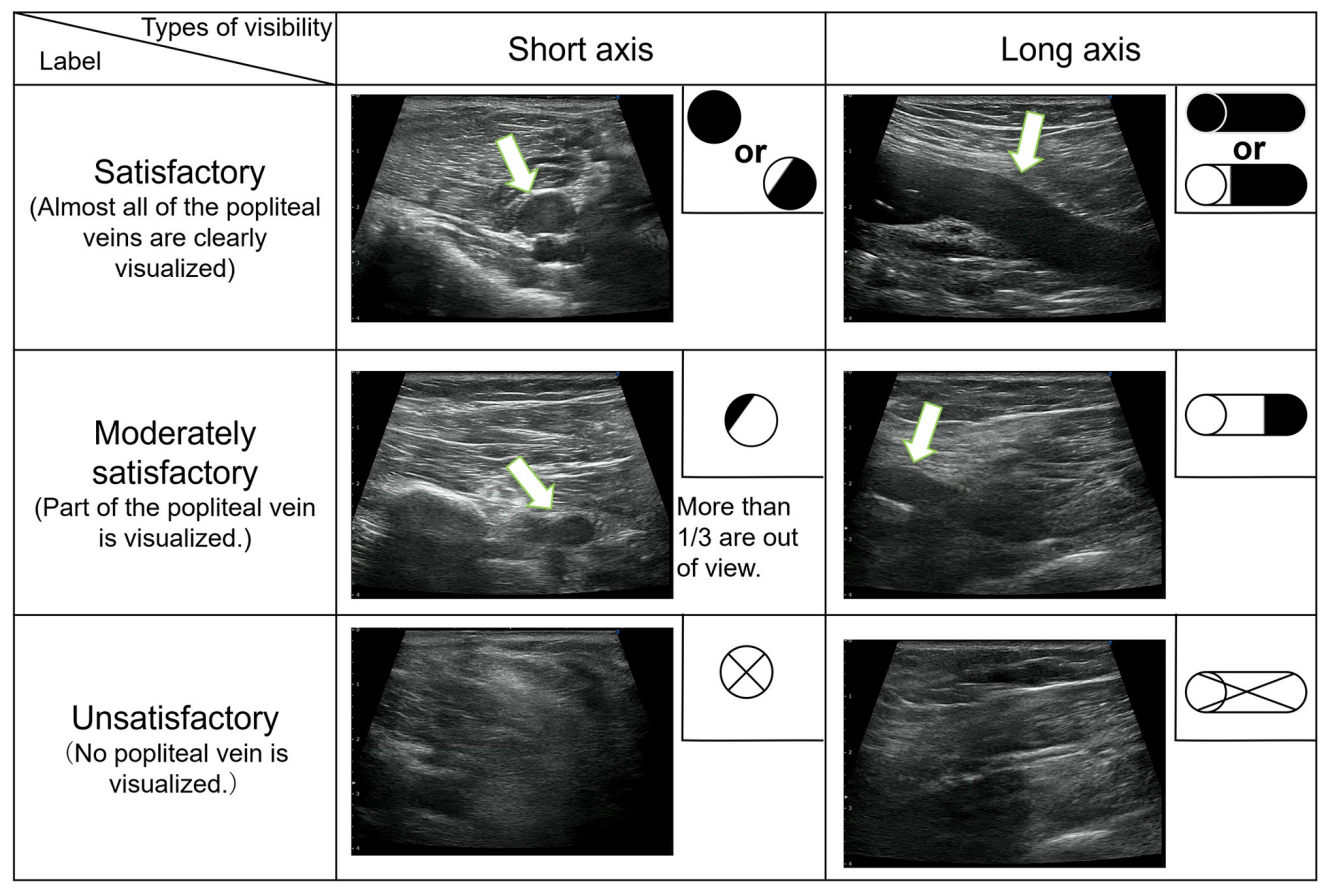
Image classification criteria defined in this study. In a schematic diagram of a blood vessel, black indicates the visualized condition, and white indicates the invisible condition.

Training data were visually labeled as "Satisfactory," "Moderately Satisfactory," or "Unsatisfactory" according to the degree that the popliteal vein was visualized on each image. An image was considered “Satisfactory” if the popliteal vein was clearly visualized. Satisfactory short-axis images were defined as images showing more than one-third of the popliteal vein. Satisfactory long-axis images were defined as images in which the popliteal vein could be visualized in at least half the length of the image. “Moderately Satisfactory” was defined as an image that could not be classified as “Satisfactory” or “Unsatisfactory.” Images were classified as “Unsatisfactory” if the popliteal veins could not be visualized. Image classification criteria are shown in Fig 2. For stationary ultrasonography, 42,837, 41,784, and 43,873 images were classified as “Satisfactory,” “Moderately Satisfactory,” and “Unsatisfactory,” respectively. For the portable ultrasonography, 13,540, 12,770, and 20,028 images were classified as "Satisfactory," "Moderately Satisfactory," and "Unsatisfactory,” respectively. These images were split into a Training dataset and a Test dataset; the Training dataset and the Test dataset used data from different cases. In other words, data from the same person were not split into a Training dataset and a Test dataset; similar images in the Training dataset were not included in the Test dataset. The images were classified by the first author, and the sonographer (sixth author) verified the classification. All images were visually verified.

The sonographic image matrix sizes were 648 × 864 pixels for the stationary ultrasound diagnostic equipment and 300 × 300 pixels for the portable ultrasound diagnostic equipment. Images were resized to 224 × 224 pixels to fit the input size of the deep learning model. Bicubic interpolation was used to interpolate the pixels.

### Deep learning and classification using fine-tuned ResNet

The runtime environment for DCNN training and classification was as follows: OS: Microsoft Windows 10; CPU: Intel Core i7-1070; GPU: NVIDIA Quadro RTX6000; Memory: 64 GB. The MathWorks MATLAB 2021a, an integrated development environment, was used for each process [15]. For DCNN, a pre-trained ResNet101 [16] was used for fine-tuning. The fully connected layer and the classification layer were re-layered to correspond to the classification of this study. ResNet101, which had already been trained by ImageNet, was used in this study [17]. The best performance for region extraction and object detection in medical images using DCNN is expected when all layers are re-trained [18, 19]. In this study, all layers were re-trained during fine-tuning. In addition, the best accuracy among other architecture selections and parameter setting were applied in this study. The parameters were set to a learning rate of 0.001, a batch size of 64, and 10 epochs. Stochastic gradient descent with momentum as an optimization algorithm was employed. In this study, the learning curve was observed to confirm that overfitting did not occur and that were learned adequately.

### Evaluation of the developed learning model

The classification accuracy of the DCNN was evaluated with 5-fold cross validation and a receiver operating characteristic curve (ROC) analysis. In this study, the probe scanning method was unified. Therefore, there were not representative for the task. In addition, all data were visually verified. Generalizability is ensured regardless which data is used. In this study, a 5-fold cross validation was performed. A description of the dataset is shown in Table 2. MathWorks MATLAB 2021a was the runtime environment for the ROC analysis. Using DCNN to classify the image, Grad-CAM [20] shows the heatmap output image of the points of interest.

**Table 2:** Description of the dataset.

Ultrasound diagnosis equipment	Satisfactory	Moderately Satisfactory	Unsatisfactory	Total
Stationary	42,837	41,784	43,873	128,494
Portable	13,540	12,770	20,028	46,338

## Results

**Fig 3:**
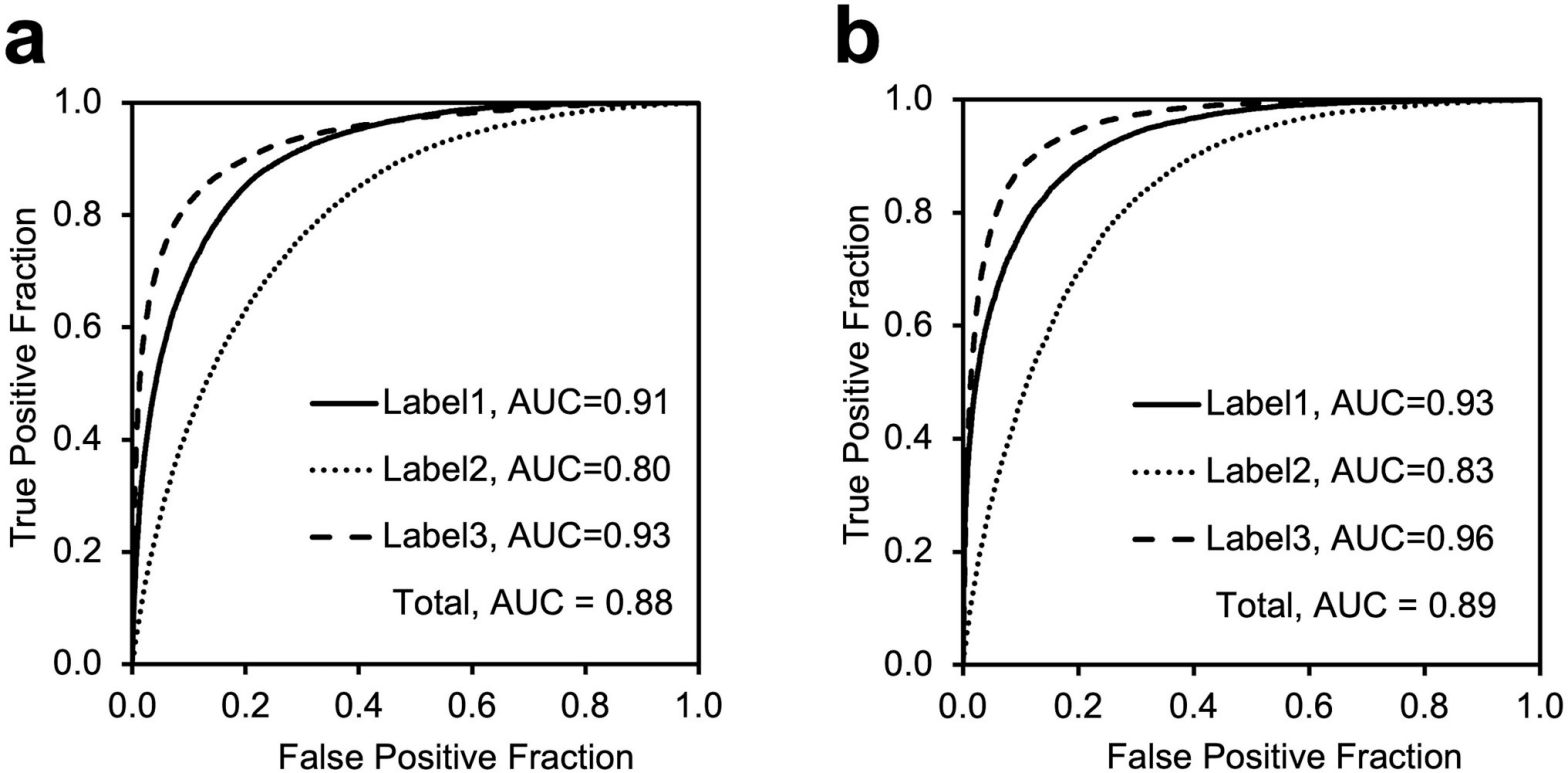
Receiver operating characteristic curves for the learned model developed in this study. (a: Stationary type, b: Portable type).

**Fig 4:**
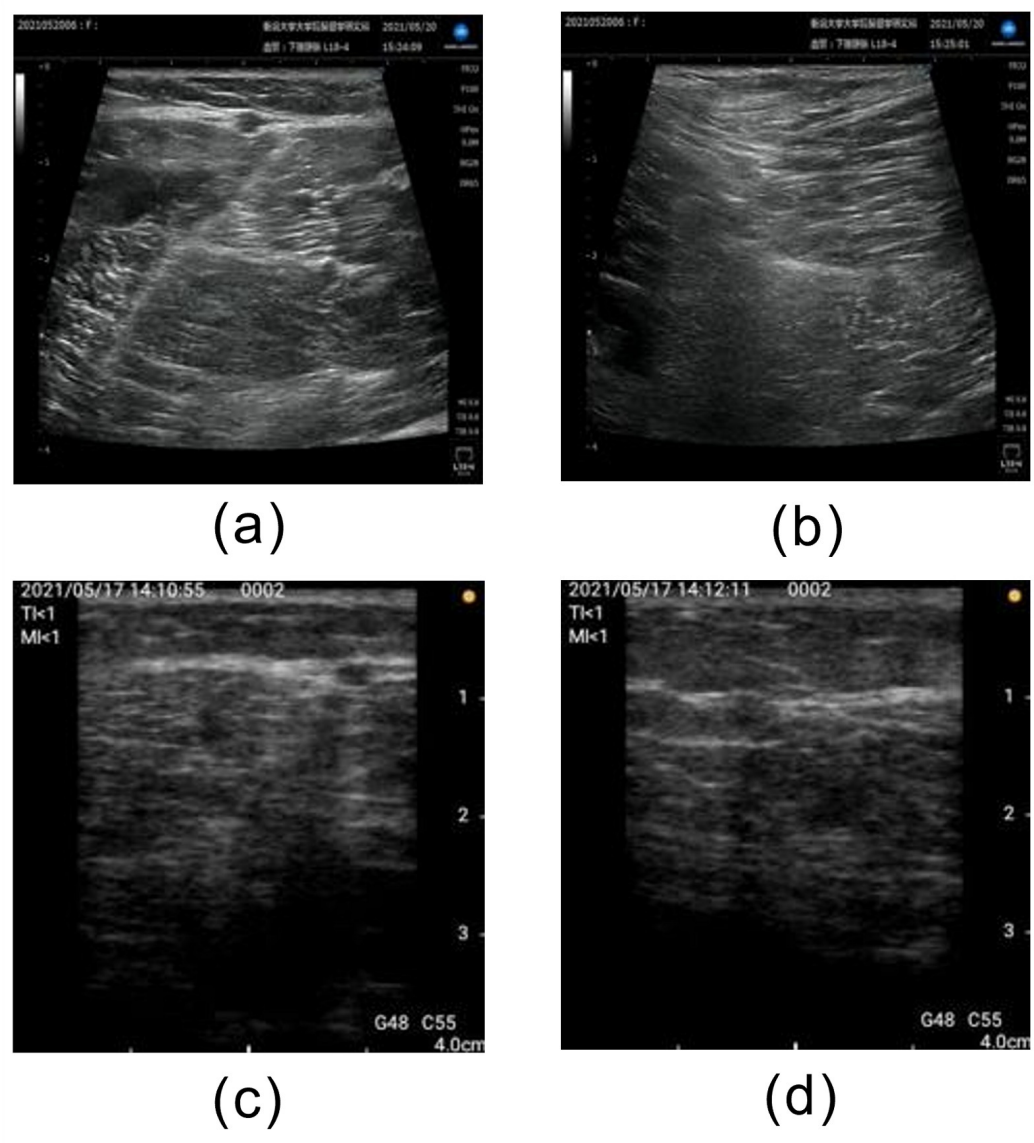
Examples of correctly classified unsatisfactory images. (a) Short axis acquired by stationary ultrasonography equipment, (b) Long axis acquired by stationary ultrasonography equipment, (c) Short axis acquired by portable ultrasonography equipment, and (d) Long axis acquired by portable ultrasonography equipment.

**Fig 5:**
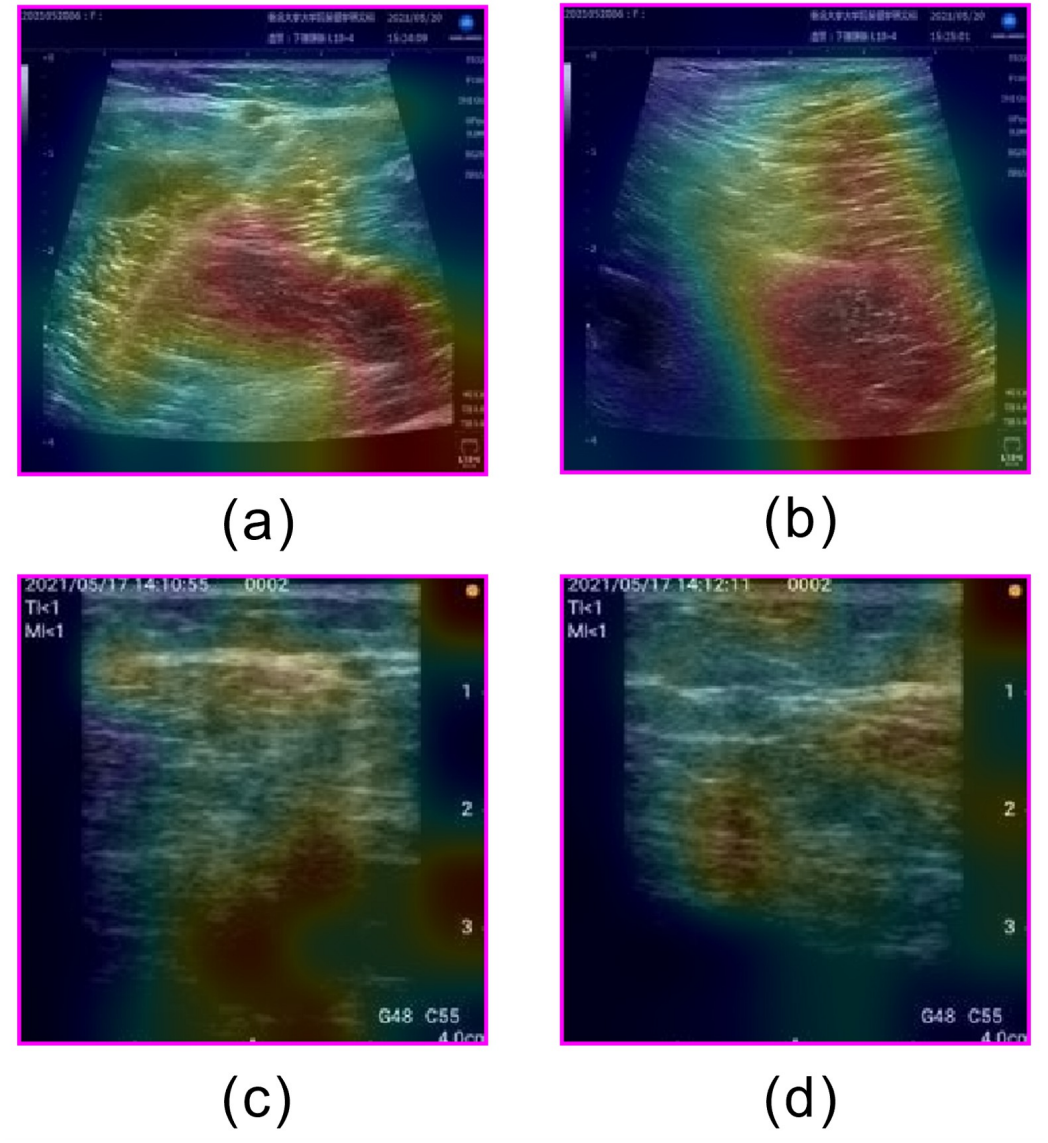
Examples of heatmaps for correctly classified unsatisfactory images. (a) Short axis acquired by stationary ultrasonography equipment, (b) Long axis acquired by stationary ultrasonography equipment, (c) Short axis acquired by portable ultrasonography equipment, and (d) Long axis acquired by portable ultrasonography equipment.

**Fig 6:**
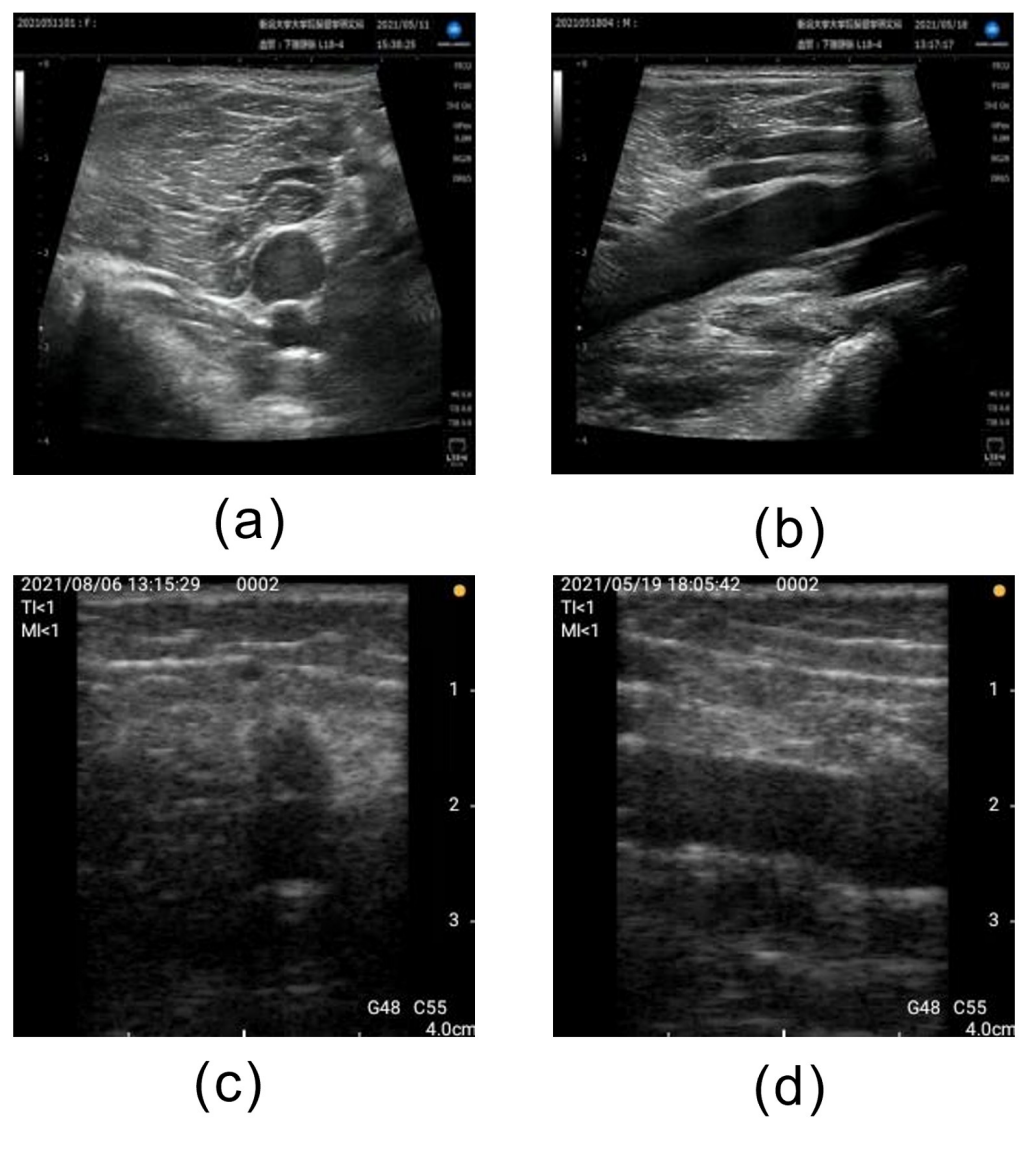
Examples of correctly classified satisfactory images. (a) Short axis acquired by stationary ultrasonography equipment, (b) Long axis acquired by stationary ultrasonography equipment, (c) Short axis acquired by portable ultrasonography equipment, and (d) Long axis acquired by portable ultrasonography equipment.

**Fig 7:**
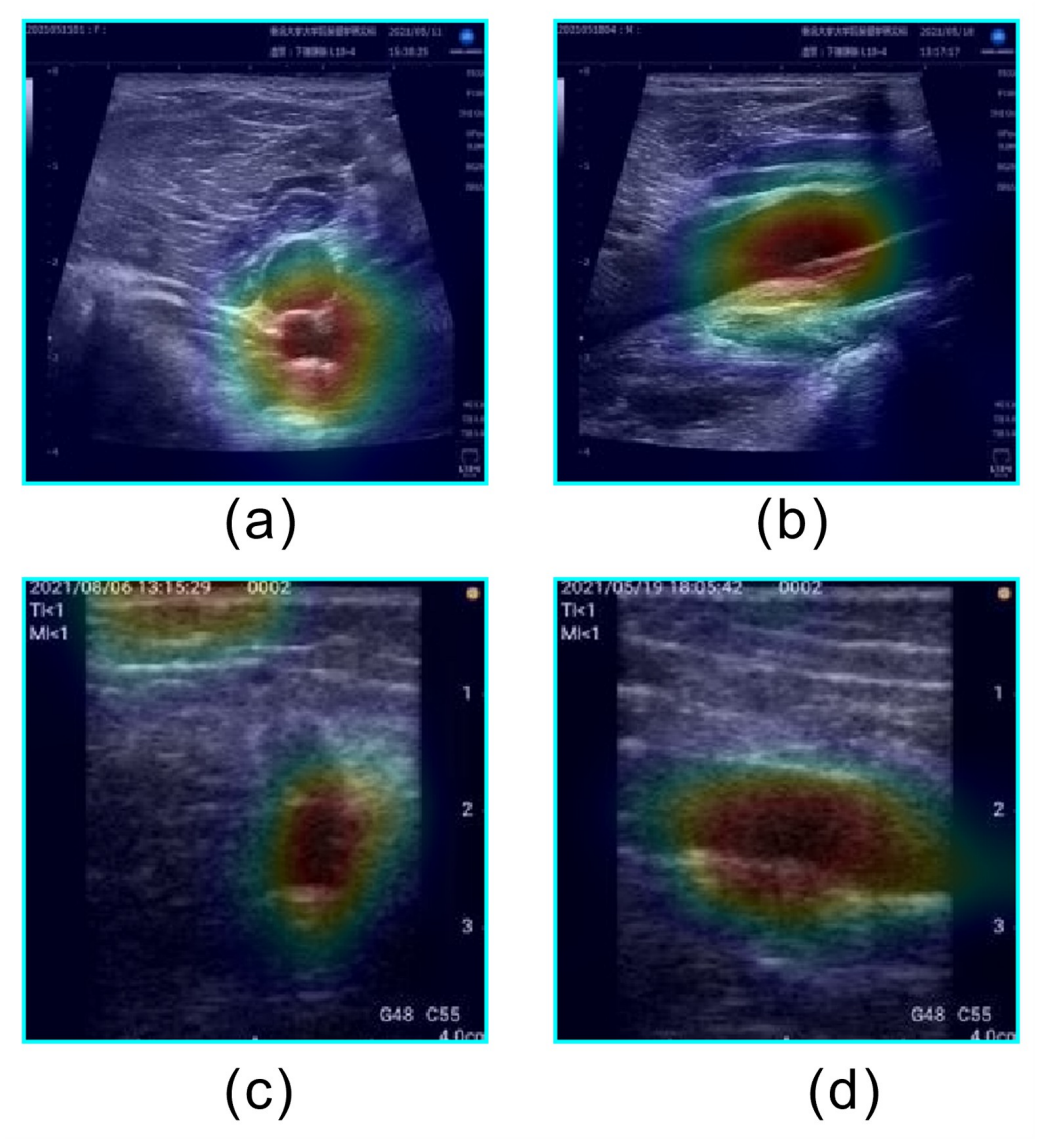
Examples of heatmaps for correctly classified satisfactory images. (a) Short axis acquired by stationary ultrasonography equipment, (b) Long axis acquired by stationary ultrasonography equipment, (c) Short axis acquired by portable ultrasonography equipment, and (d) Long axis acquired by portable ultrasonography equipment.

**Fig 8:**
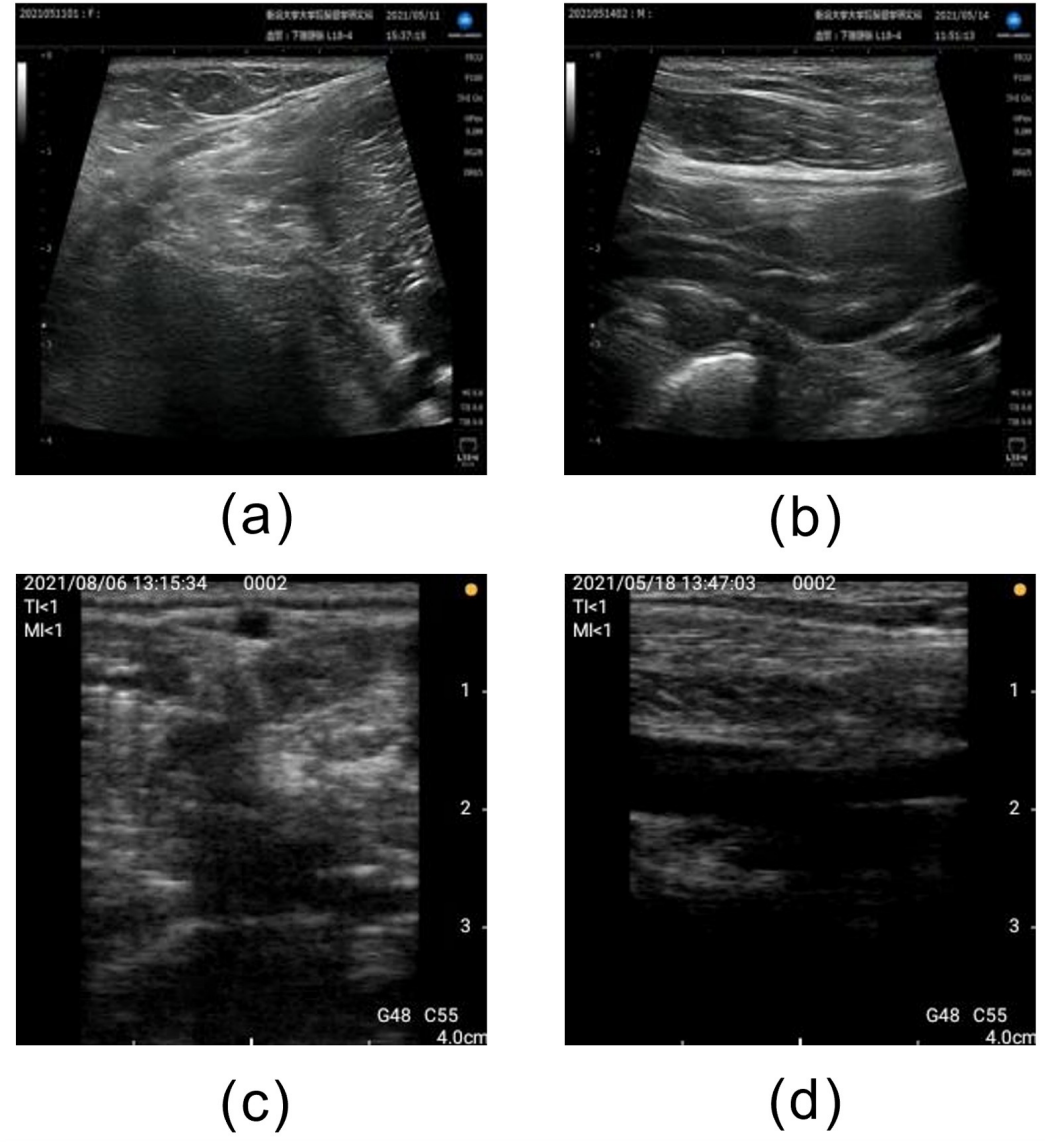
Examples of correctly classified Moderately Satisfactory images. (a) Short axis acquired by stationary ultrasonography equipment, (b) Long axis acquired by stationary ultrasonography equipment, (c) Short axis acquired by portable ultrasonography equipment, and (d) Long axis acquired by portable ultrasonography equipment.

**Fig 9:**
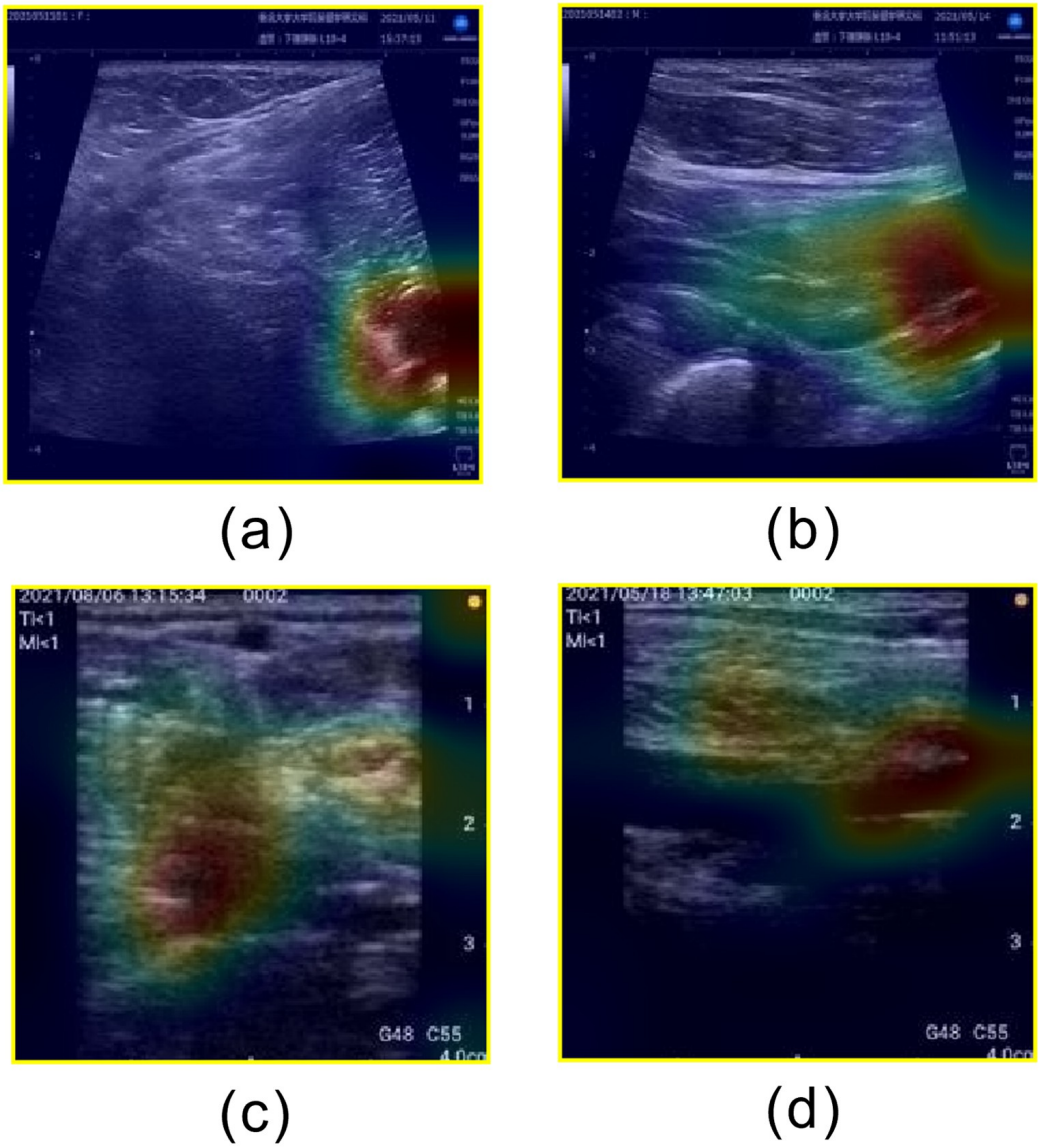
Examples of heatmaps for correctly classified Moderately Satisfactory images. (a) Short axis acquired by stationary ultrasonography equipment, (b) Long axis acquired by stationary ultrasonography equipment, (c) Short axis acquired by portable ultrasonography equipment, and (d) Long axis acquired by portable ultrasonography equipment.

The ROC curves are shown in Fig 3a and 3b. The total area under the ROC (AUC) was 0.88 for stationary ultrasonography and 0.89 for portable ultrasonography. For the stationary ultrasonography, the AUCs for Satisfactory, Moderately Satisfactory, and Unsatisfactory were 0.91, 0.80, and 0.93, respectively. For the portable ultrasonography, the AUCs for Satisfactory, Moderately Satisfactory, and Unsatisfactory were 0.93, 0.83, and 0.96, respectively. Tables 3 and 4 show the confusion matrixes for the stationary and portable types, respectively. The classification accuracies, calculated from the confusion matrixes, are shown in Table 5. Using the images acquired from the stationary ultrasound diagnostic equipment, the classification accuracy was 0.73 (93,241/128,494). Using the images acquired from the portable ultrasound diagnostic equipment, the classification accuracy was 0.76 (35,444/46,338). Correctly classified images for each label are shown in Figs 4, 6 and 8. The images with a heatmap of the area representing the basis of the classification are shown in Figs 5, 7 and 9.

**Table 3:** Normalized confusion matrix for stationary ultrasonography.

Predict			
True	Satisfactory	Moderately Satisfactory	Unsatisfactory
Satisfactory	0.74 (31,702/42,837)	0.23 (9,681/42,837)	0.03 (1,454/42,837)
Moderately Satisfactory	0.22 (9,228/41,784)	0.61 (25,616/41,784)	0.17 (6,940/41,784)
Unsatisfactory	0.03 (1,185/43,873)	0.15 (6,765/43,873)	0.82 (35,923/43,873)

Normalized number of classified are as follows; Total number of correctly classified images in 5-fold cross validation / Total number of images in the class.

**Table 4:** Normalized confusion matrix for portable ultrasonography.

Predict			
True	Satisfactory	Moderately Satisfactory	Unsatisfactory
Satisfactory	0.73 (9,883/13,540)	0.23 (3,175/13,540)	0.04 (482/13,540)
Moderately Satisfactory	0.20 (2,553/12,770)	0.60 (7,723/12,770)	0.20 (2,494/12,770)
Unsatisfactory	0.01 (193/20,028)	0.10 (1,997/20,028)	0.89 (17,838/20,028)

Normalized number of classified are as follows; Total number of correctly classified images in 5-fold cross validation / Total number of images in the class.

**Table 5:** Classification accuracy using the learned model.

Ultrasound diagnosis equipment	Satisfactory	Moderately Satisfactory	Unsatisfactory	Total
Stationary	0.74 (31,702/42,837)	0.61 (25,616/41,784)	0.82 (35,923/43,873)	0.73 (93,241/128,494)
Portable	0.73 (9,883/13,540)	0.60 (7,723/12,770)	0.89 (17,838/20,028)	0.76 (35,444/46,338)

Normalized number of classified are as follows; Total number of correctly classified images in 5-fold cross validation / Total number of images in the class.

## Discussion

DVT is a public health problem and the risk of DVT increases after disasters. The early prevention of DVT can be achieved by various approaches. Tanno et al. [14] developed an automated ultrasonography system for the accurate detection of DVT consisting of a method to automatically detect vein regions using deep learning and a method to automatically determine whether the veins are properly visualized. Smistad et al. [13] proposed a method to automatically detect blood vessels in an image using a geometric mathematical blood vessel model and deep learning. The objective of the current study was to develop a DVT risk assessment for disaster victims by automating venous ultrasonography of the lower extremity. For the development of this system, a method that enables the victim to use the ultrasound diagnostic equipment and acquire images visualizing popliteal veins was necessary before applying the automatic vascular information acquisition system of Tanno et al. and Smistad et al. We developed a method that can automatically classify cross-sectional images suitable for diagnosis during venous ultrasonography of the lower extremities, even for disaster victims who have no experience in ultrasonography.

The AUCs for this method were 0.87 and 0.89 using stationary and portable diagnostic ultrasound equipment, respectively. Classification accuracies of 0.72 and 0.76 were achieved for the stationary and portable diagnostic ultrasound equipment, respectively. Both the stationary and portable equipment showed the highest AUCs for Unsatisfactory classifications and the lowest AUCs for Moderately Satisfactory classifications. Fig 4 shows an example of a correctly classified “Unsatisfactory” image. The images classified as "Unsatisfactory" showed no popliteal veins and no parallel lines or circles characteristic of popliteal veins. We believe that the wide area of the image is the basis for classification and the popliteal vein is judged to be non-existent in the image. Furthermore, although superficial veins are present in the upper part of the images in Fig 5a and 5c, they do not provide a basis for classification. Therefore, we believe that the learned model developed in this study is based on the presence or absence of popliteal vein visualization. Fig 6 shows an example of an image that was correctly classified as "Satisfactory" with a high ACC, and Fig 7 shows the heatmap for this image. The image classified as "Satisfactory" clearly shows popliteal veins.

The learned model developed in this study focuses on the popliteal vein for classification, as shown in Fig 7. The ability to correctly classify "Satisfactory" images is important because the popliteal vein must first be properly visualized to assess the risk of incidental DVT. Our newly developed method allows the victim to confirm the "Satisfactory" indication and acquire the image. The high ACC for "Satisfactory" images indicates the effectiveness of the learned model in automatically identifying images with popliteal veins. Furthermore, Table 3 shows that the most frequently misclassified label in the Satisfactory data set was Moderately Satisfactory. Furthermore, the most frequently misclassified label in the Unsatisfactory data set was Moderately Satisfactory. The catastrophic misclassification in this guide system is the misclassification of Unsatisfactory images as Satisfactory. Because most misclassifications in this study were Moderately Satisfactory, catastrophic misclassifications tended to be small.

Figs 8 and 9 show examples of correctly classified “Moderately Satisfactory” images, which showed the lowest AUC, and the corresponding heatmap images. “Moderately Satisfactory” indicates an image that cannot be judged as either “Satisfactory” or “Unsatisfactory.” Therefore, visually classifying an image as “Moderately Satisfactory” is difficult relative to classifying “Satisfactory” images, which exhibit clearly visible popliteal veins, or “Unsatisfactory” images in which the popliteal vein is not visible. The basis for judging “Moderately Satisfactory” was more difficult to learn than the basis for the other labels. For this reason, “Moderately Satisfactory” had the lowest AUC. However, the low classification accuracy of “Moderately Satisfactory” is not a problem for this method of DVT risk assessment. For this method, the operator scans the probe from “Unsatisfactory” to “Moderately Satisfactory” and then to “Satisfactory” to obtain images that are optimal for diagnosis. In actual use, the victims first put the probe on the lower extremity. Then, the labels “Satisfactory,” “Moderately Satisfactory,” or “Unsatisfactory” appears on the screen. If the victim is not experienced in ultrasonography, the probe cannot hit the appropriate vein, and “Unsatisfactory” will be displayed. “Moderately Satisfactory” is indicated during the process of scanning various parts of the lower extremity. The “Moderately Satisfactory” display indicates that there is a location near the scanning site where the popliteal vein can be clearly visualized. A detailed scan around the "Moderately Satisfactory" area eventually results in clear visualization of the popliteal vein and "Satisfactory" is indicated. Therefore, the "Moderately Satisfactory" classification functions to make the operator aware of the vicinity of the popliteal vein in the image and is not directly involved in the acquisition of the best cross-sectional image for diagnosis. Thus, the accuracy of "Moderately Satisfactory" is not considered important.

As mentioned above, in this study, a guide system was developed to enable the victims themselves to use the ultrasound diagnostic equipment. The system indicates the level of clarity of the current vein visualization, allowing victims with no ultrasound experience to be easily guided to a fully visualized vein. The two-class classification of Unsatisfactory and Satisfactory labels takes extra time to display the Satisfactory image because it is not possible to determine whether the probe is close to a position where the vessel can be clearly visualized or whether the probe is in a completely misplaced position. In this study, the Moderately Satisfactory label was defined, which intermediate between the two labels. With this approach, we believe that it is possible to ensure the allowance until the veins are fully visualized.

**Fig 10:**
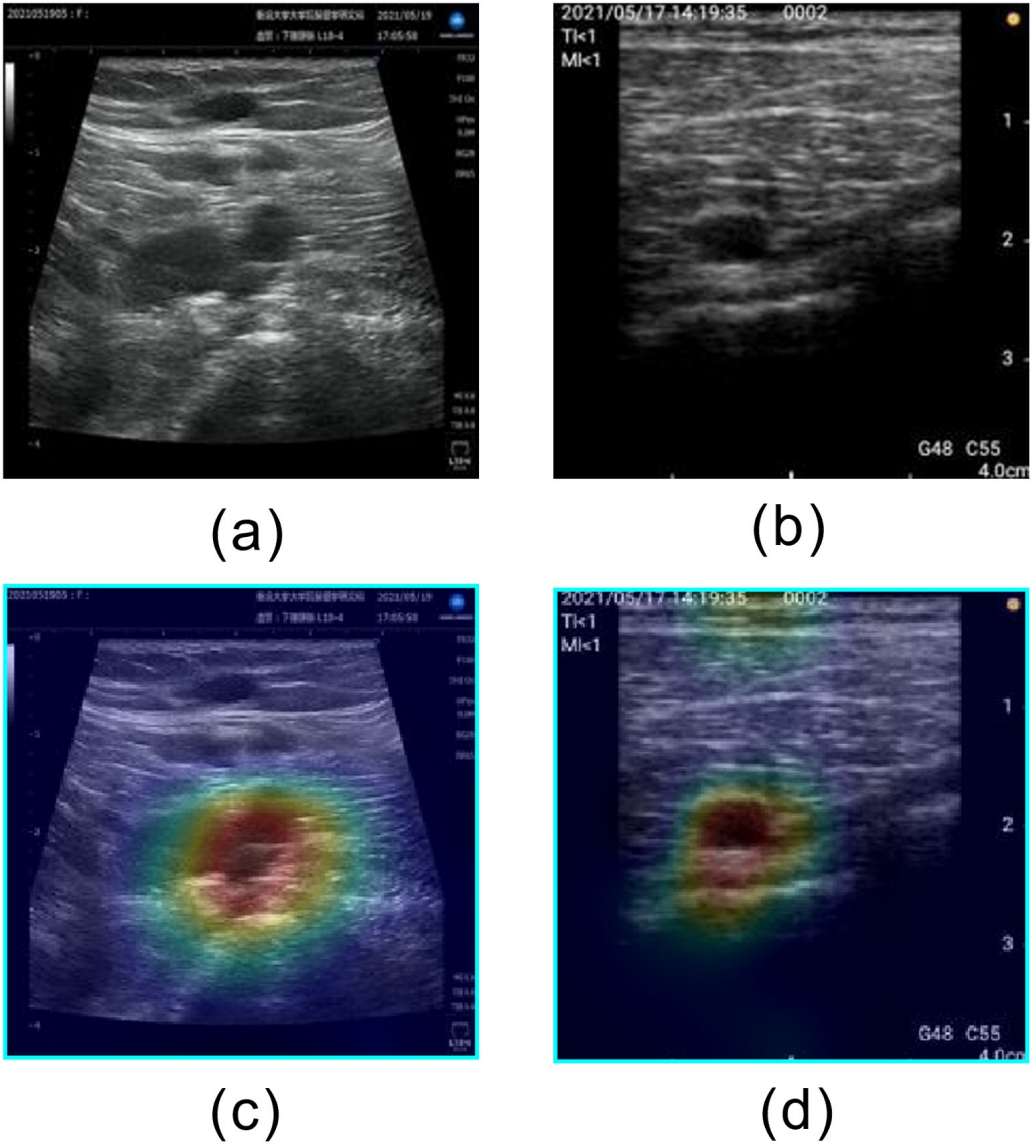
Examples of Moderately Satisfactory images misclassified as satisfactory images and the corresponding heatmap images. (a) Long axis acquired by stationary ultrasonography equipment, (b) Long axis acquired by Portable ultrasonography equipment, (c) Long axis acquired by stationary ultrasonography equipment, and (d) Long axis acquired by portable ultrasonography equipment.

Examples of misclassified images and their heat maps are shown in Fig 10. Some of the long-axis images were judged as short-axis images because we trained a mixed dataset of short-axis and long-axis images. The accuracy could be improved by increasing the number of cases to be trained in the future. We focused on the popliteal vein in this study. Further validation is required to confirm similar accuracy in the soleal and iliac veins [1], which are the preferred sites for DVT in venous ultrasonography in addition to the popliteal.

We developed a deep learning model that classifies popliteal veins based on their visual clarity. By attaching color frames based on the classification results, the victim can intuitively recognize the appearance of veins on the image through visual information, which will assist the victim in scanning. Although the AUC for "Moderately Satisfactory" was low, the overall AUC and the AUC for "Satisfactory,” which is directly related to the automatic diagnosis, were high. Our results indicate that this method can be used to automatically acquire cross-sectional images suitable for diagnosis by venous ultrasonography of the lower extremities without depending on the victim’s experience with ultrasonography.

In this study, all training and validation data were acquired from healthy persons. Further investigation is needed to determine whether the deep learning model developed in this study can be performed with almost same level of accuracy in cases using images with DVT.

## Conclusions

A method for automatic identification of appropriate cross-sectional images for diagnosis from ultrasonographic images of the popliteal vein was developed using fine-tuned ResNet-101. Ultrasonographic images of the popliteal veins of 20 subjects acquired from both stationary and portable ultrasound diagnostic equipment were automatically classified into three categories according to the level of vein visibility. As a result, 73% classification accuracy was achieved using stationary ultrasound diagnostic equipment, and 76% classification accuracy was achieved using portable ultrasound diagnostic equipment. Although more improvement is needed, the total AUC and the AUC for “Satisfactory” images, which are linked to the automatic diagnosis of DVT, were high. Our results indicate that this method can be used to automatically identify the appropriate ultrasonographic cross-sectional image for the diagnosis of DVT using venous ultrasonography of the lower extremity, without relying on the victim’s ultrasonography skills.
